# Investigation on the Mechanism and Failure Mode of Laser Transmission Spot Welding Using PMMA Material for the Automotive Industry

**DOI:** 10.3390/ma10010022

**Published:** 2017-01-01

**Authors:** Xiao Wang, Baoguang Liu, Wei Liu, Xuejiao Zhong, Yingjie Jiang, Huixia Liu

**Affiliations:** School of Mechanical Engineering, Jiangsu University, Zhenjiang 212013, China; liubaoguang103@163.com (B.L.); liuweiwei2964@163.com (W.L.); zhongxj66@163.com (X.Z.); 18852868861@163.com (Y.J.); lhx@ujs.edu.cn (H.L.)

**Keywords:** laser technique, laser transmission spot welding, welding mechanism, thermoplastic polymer, morphology of weld pool

## Abstract

To satisfy the need of polymer connection in lightweight automobiles, a study on laser transmission spot welding using polymethyl methacrylate (PMMA) is conducted by using an Nd:YAG pulse laser. The influence of three variables, namely peak voltages, defocusing distances and the welding type (type I (pulse frequency and the duration is 25 Hz, 0.6 s) and type II (pulse frequency and the duration is 5 Hz, 3 s)) to the welding quality was investigated. The result showed that, in the case of the same peak voltages and defocusing distances, the number of bubbles for type I was obviously more than type II. The failure mode of type I was the base plate fracture along the solder joint, and the connection strength of type I was greater than type II. The weld pool diameter:depth ratio for type I was significantly greater than type II. It could be seen that there was a certain relationship between the weld pool diameter:depth ratio and the welding strength. By the finite element simulation, the weld pool for type I was more slender than type II, which was approximately the same as the experimental results.

## 1. Introduction

In recent years, with the increasingly severe issues of global resources and environmental protection, innovative research of lightweight products for energy savings and environmental protection is becoming a new trend of development. Especially in the automotive industry, researchers are working to reduce the weight of the body for alleviating the problem of energy consumption and tail gas pollution caused by the growing number of cars. Thermoplastic polymer materials have the advantages of being light weight, having a high level of strength, low density, easy molding, low cost, good flexibility, corrosion resistance, etc. [1,2]. Therefore, the thermoplastic polymer materials become the first choice for replacing steel and cast iron.

Spot welding technology, as a kind of high efficient polymer connection technology, has been widely used in automobile manufacturing [3]. The traditional methods of spot welding include friction stir welding, ultrasonic spot welding, friction lap joining, etc. [4,5,6]. Ultrasonic welding, now an important welding method, has the best welding precision. However, there are limitations due to it being contact welding. Mustafa et al. [4] studied the process parameters of friction stir welding using high-density polyethylene (HDPE). The depth, velocity and time of rotation was controlled, the new theory of the Taguchi method was used to optimize the experimental parameters. Jeng et al. [7] studied the relationship between different ultrasonic spot welding parameters and welding quality. The research also found that the temperature rise during the welding process also affected the initial bonding strength of the welding parts. Paoletti et al. [8] analyzed the force and torque developing during friction stir spot welding (FSSW) of thermoplastic sheets varying the main process parameters. According to the achieved results, using low values of the plunging speed has beneficial effects on both the process (reduction in the force and torque) and the mechanical behaviour of the joints. Increasing the tool rotational speed results in reduced processing forces and higher material mixing and temperature. Jeng et al. [9] studied polycarbonate sheets in order to assess the influence of the tool geometry on the joining loads and material flow by friction stir spot welding. An instrumented drilling machine was used to measure the plunging load and the torque developing during the process. The analysis of material flow enabled understanding the behavior of the load and torque trends measured during the process. Okada et al. [6] proposed a Friction Lap Process to join a metallic material with a polymer and investigated mechanical and metallurgical properties of this dissimilar joint. In this paper, the joining mechanism was discussed with evaluation of the microstructure at the interface between aluminum alloy and polymer. Lambiase et al. [10] analyzed the influence of the processing speeds and processing times on mechanical behaviour of friction stir spot welding joints produced on polycarbonate sheets. The analysis involved the variation of rotational speed, tool plunge rate, pre-heating time, dwell time and waiting time. Compared to the traditional polymer spot welding technology, laser spot welding is non-contact welding and has the advantages of high welding quality, being eco-friendly, having a small heat affected zone, etc. [11,12]. In the laser spot welding technology, laser transmission spot welding uses the light transmissions of thermoplastics to weld components. Thus, the laser transmission spot welding is the most potential welding method in the trend of substituting steel for plastic, and this technology is gradually applied to all walks of life.

In recent years, the research on laser transmission spot welding of polymers is rarely involved. Visco et al. [12] used an Nd:YAG pulsed laser welding of polymers to optimize the welding parameters, and the experiment mainly focused on the single factor optimization regarding the action time. Yusof et al. [13] studied the welding performance on laser transmission welding of plastics with different metals using microscopic observations and tensile tests. The difference of the connection strength between different welding materials was obtained by controlling the welding parameters. However, there are few studies on the mechanism and failure mode of laser transmission spot welding, especially for the laser transmission spot welding of polymers. Lambiase et al. [14] investigated Laser-Assisted Metal and Plastic bonding (LAMP) of AISI304 sheets with polycarbonate sheets, which introduced an integrated experimental approach aimed at understanding how the main process conditions influence welding quality, dimensions and presence of defects. With the development of society, the lack of scientific and reasonable connection mechanism analysis will affect the application of laser transmission spot welding technology in the industry

In this paper, the widely used thermoplastic polymer polymethyl methacrylate (PMMA) in the automotive industry was chosen as the research object [15]. The Nd:YAG pulse laser with a wavelength of 1064 nm was used to weld materials during laser transmission spot welding. This research mainly adopted two kinds of welding types. Through the comparative study of the bubble and the failure mode, the welding mechanism was further understood. Then, through the optical microscope observation of the weld pool, the weld pool diameter:depth ratio corresponding to two welding types was analyzed, which helped us understand the effect of the melt pool diameter:depth ratio on the welding strength. Finally, the influence of laser energy density on the welding pool was obtained by the finite element simulation. The welding pool for different welding types was compared and analyzed.

## 2. Materials and Methods

In this experiment, the upper and lower materials are thermoplastic polymers PMMA, and the sample size is 50 mm × 20 mm × 1.5 mm. The research adopted the Nd:YAG pulsed laser with a wavelength of 1064 nm and the peak power was 7 kW (Rofin, München, Germany), which can directly set up peak voltages and frequency et al. In the laser transmission welding process, the upper material must be transparent. The underlying material should have higher absorption properties [16]. The transparent material must have good transparency. The transmittance of PMMA in the wavelength of 1064 nm was 86%, measured by ultraviolet visible near infrared absorption spectroscopy (Spectrograph Type Cary 5000, Varian Corporation, Palo Alto, CA, USA). Therefore, it was necessary to add absorbent Clearweld (c/o Crysta-Lyn Chemical Company, Inc., Binghamton, NY, USA) on the surface of the lower layer material in the experiment. Since the amount of absorbent in the welding process was also an important factor, the amount of absorbent Clearweld painted on each sample should remain approximately the same.

**Figure 1:**
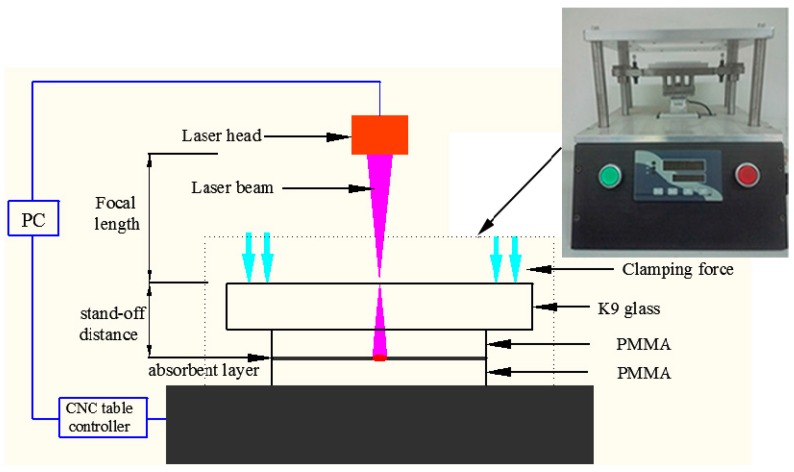
Schematic diagram of a laser transmission spot welding experiment device.

During the experiment, the pneumatic clamping device was adopted to ensure the uniformity of the clamping force in the process of laser transmission spot welding. After many experiments, the clamping force of 20 N is a relatively ideal value. The K9 glass was used as the clamping layer. The schematic diagram of the experimental device of laser transmission spot welding was shown in Figure 1.

In this experiment, the pulse width of the laser was 2 ms for all welded samples, the pulse frequency’s duration was 25 Hz. In addition, 0.6 s was recorded as type I and pulse frequency, and the duration was 5 Hz. Furthermore, 3 s was recorded as type II (see Table 1), the pulse frequency was reduced five times and duration increased five times. Two parameters were varied: peak voltage and defocusing distance. Three peak voltages (440 V, 460 V, 480 V) and three defocusing distances (6 mm, 8 mm, 10 mm) were studied.

After welding, the tensile test was carried out by the microcomputer control electronic universal testing machine (Instron Type UTM 4104, Shenzhen, China). The maximum tensile strength of the two welding methods were obtained and analyzed. In order to get better results for the tensile tests, the tensile speed should try to be controlled at less than 0.5 mm/min during shear tensile experiments. Then, the microstructure of the solder joints was observed by Kean’s electron microscope (Keyence Corporation, Osaka, Japan), and the cross section morphology of the solder joints was analyzed under the two welding methods. The weld depth and weld diameter were measured by a polarizing microscope (Axio Lab.A1 pol, Carl Zeiss, Oberkochen, Germany). The relationship between the weld diameter:depth ratio and the maximum tensile strength was obtained. Finally, the influence of laser energy density on the welding temperature was obtained by finite element simulation. The weld pool in different welding types is compared and analyzed.

## 3. Results and Discussion

### 3.1. The Effect of Bubbles in the Spot Welding Area

**Figure 2:**
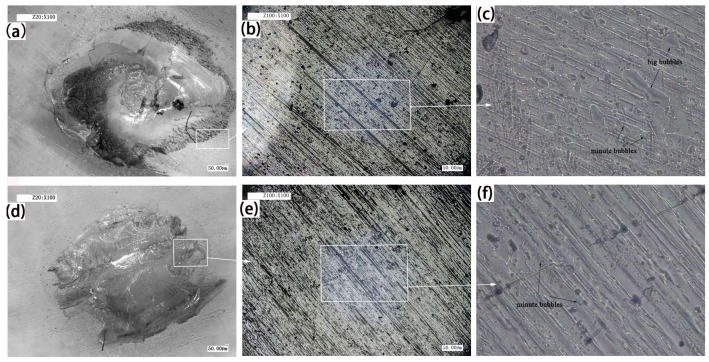
Weld morphology of type I, 460 V, 8 mm. (**a**) Magnified 50 times; (**b**) magnified 100 times; (**c**) magnified 500 times. Weld morphology of type II, 460 V, 8 mm; (**d**) magnified 50 times; (**e**) magnified 100 times; and (**f**) magnified 500 times.

Laser transmission spot welding is a kind of hot melt welding, and the polymer absorbs some energy and converts it into heat, so that the upper layer and lower layer material can be melted and mixed to form the weld. However, during the welding process, the heating zone often produces bubbles and ablation due to defects such as welding conditions and process, which affects the appearance and welding performance. Figure 2 shows the welding morphology of PMMA in the type I, 460 V, 8 mm and type II, 460 V, 8 mm. Figure 2b,e shows the weld morphology of type I, 460 V, 8 mm and type II, 460 V, 8 mm when the magnification is 100 times. A certain difference in the morphology between the two analyzed cases is appreciable in the higher magnification pictures. A small number of large bubbles and many small bubbles can be seen in Figure 2c. For the high input laser energy density, the degradation and bubbles occur in the weld. Meanwhile, the short action time leads to the escape of bubbles, and it is easy to generate many bubbles [17]. For the same peak voltage and defocusing distance, long laser action time and low pulse frequency make the molten pool of small bubbles have enough time to converge and escape, which leads to some very small bubbles occurring, as shown in Figure 2f. In the process of thermal degradation of plastics, these bubbles mainly consist of water vapor, carbon dioxide, carbon monoxide, and hydrocarbons [18]. These tiny bubbles will cause the material surface to generate pits and cracks, which can trigger the micro-anchor mechanism and increase the joining strength. Liu et al. [19] studied the friction lap welding between the aluminum alloy and polyethylene glycol terephthalate, and they found that these bubbles produce high pressure and make the fused plastic flow onto the pits or holes of the metal surface, so as to provide more mechanical binding to achieve a tight connection between metals and polymers. Therefore, to a certain extent, the bubbles have certain benefits to improve the welding quality.

### 3.2. Shear Failure Analysis

**Figure 3:**
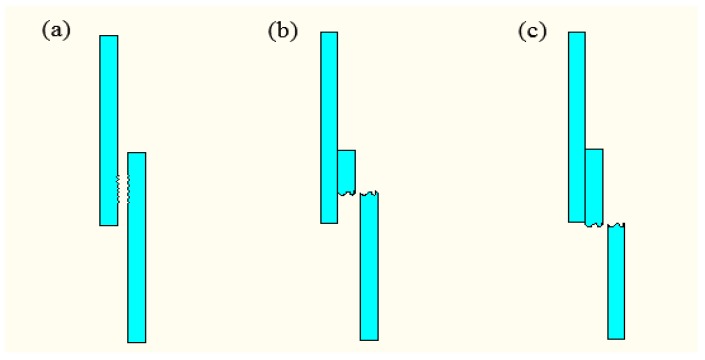
Failure modes of plastic welding parts. (**a**) Interface failure; (**b**) the base sheet fracture along the welding joint; (**c**) bulk base sheet fracture

Failure analysis plays a very important role and is significant in the manufacturing of mechanical parts. Through the failure analysis, it is helpful to formulate the process parameters reasonably and improve the adaptability of the application [20]. During the tensile shear tests, the failure modes of plastic welding parts are shown in Figure 3, which have been adapted from classifications used with adhesive-based joints [21,22]. There are two main types of failure: interfacial and substrate. The failure of the interface is mainly due to the tensile strength of the welded joint, which is smaller than that of the base plate. When the welding intensity is getting larger, the fracture will generate along the weld seam and even the center of the base plate will be broken.

**Figure 4:**
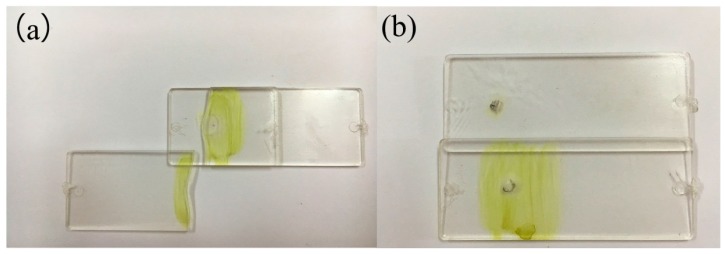
The fracture modes of 480 V, 6 mm. (**a**) type I; (**b**) type II.

In order to analyze the different fracture modes of plastic welding parts, the pulse voltage used in the experiments was 480 V, and the defocusing distance was 6 mm. The morphology changes of the welding spots after shearing tests were observed by the KEYENCEVHX-1000C digital microscope (Keyence Corporation). Under the above-mentioned process parameters (480 V, 6 mm), Figure 4 shows the two different fracture forms of plastic welding parts, namely type I and type II, which were conducted by using a controlled electronic universal testing machine (Instron Type UTM 4104, Shenzhen, China). Due to the welding strength being higher than the tensile strength of the base plate, the failure mode of type I is the base plate fracture that generates along the welding joint. However, the bonding strength of type II is obviously smaller than that of type I, which results in the interfacial failure. Then, further analysis has been carried by the KEYENCEVHX-1000C digital microscope.

**Figure 5:**
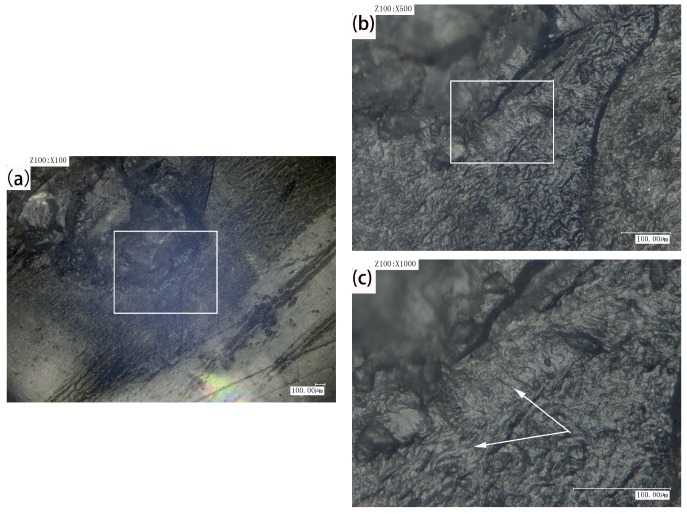
Post-failure images of the bond interface for the samples (type II, 480 V, 6 mm), (**a**) magnified 100 times; (**b**) magnified 500 times; (**c**) magnified 1000 times.

Figure 5 shows post-failure images of the bond interface for the samples (type II, 480 V, 6 mm), and the tensile failure mode is interface failure. As shown in Figure 5a, there is an obvious ablation phenomenon in the central area of laser irradiation, which is caused by the high density of energy input. Materials will become more brittle in the ablation region, where it is easy to produce the brittle fracture phenomenon. Nevertheless, the energy input density becomes smaller at the edge of the weld area, and the pyrolysis effect becomes weaker. At the same time, the diffusion and entanglement of the upper and lower molecular chains become stronger, which can produce obvious local ductile tearing, as shown in Figure 5b,c. During the stretching process, the fracture surface of the sample is a mixed type of fracture including the brittle fracture and ductile fracture. According to the measuring results, the maximum load is about 268 N.

**Figure 6:**
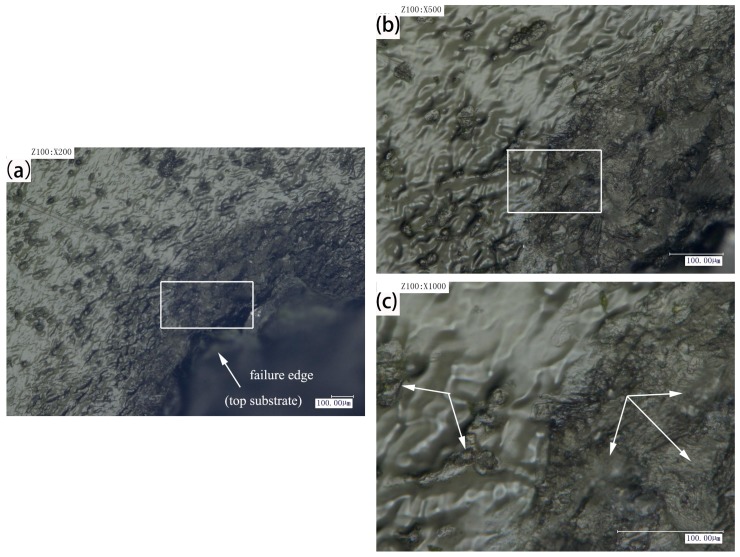
Post-failure images of the bond interface for the samples (type I, 480 V, 6 mm), (**a**) magnified 200 times; (**b**) magnified 500 times; (**c**) magnified 1000 times.

Figure 6 shows post-failure images of the bond interface for the samples (type I, 480 V, 6 mm). As can be seen from Figure 6a, it is obvious that tensile failure mode is the base sheet fracture along the welding joint, which shows that the welding strength is very high, even higher than the tensile strength of the substrate. Figure 6a is the failure morphology of welded sample, when the magnification is 200 times. The phenomenon of tensile deformation cannot be seen in this figure. However, it can be seen that there are a lot of bubbles generated. Furthermore, the number and size of the bubbles are closely related to the peak voltage and pulse frequency. These bubbles make the upper and lower layers of the material achieve micro riveting, which increases the welding strength. From Figure 6b,c, due to the large laser energy density, the serious ductile deformation and the local brittle fracture occur in the specimen during the tensile process. The failure of the materials can be attributed to the stress concentration induced by bubbles, ablation and other defects during the welding process. The unbalanced stress was caused by delamination in the welding seam. However, owing to the materials being melted and fully combined, intense action occurs in the molecular chain. The maximum load of the welding joint is higher, which is about 350 N.

### 3.3. The Effects of Pulsed Laser Parameters on the Weld Dimensions

**Figure 7:**
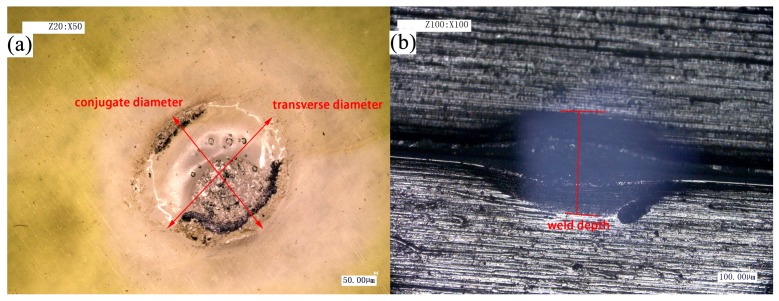
Morphology of weld pool (**a**) weld diameter; (**b**) weld depth.

The heat-affected zone (HAZ) is well defined in the welding of metals. It is the region of the material near the weld where property and microstructural changes occur as a result of heat conduction from the weld seam [23]. An HAZ can also occur in polymer welding [24,25]. In order to analyze the relationship between welding strength and weld diameter:depth of weld in two forms, the KEYENCEVHX-1000C digital microscope (Keyence Corporation) was used to observe the microstructure of the weld pool. The morphology of the weld pool is shown in Figure 7. When measuring the diameter of the weld pool, due to the irregularity of the welding joint, the weld diameter is calculated as:

weld diameter = (transverse diameter + conjugate diameter)/2.(1)

The parameters of weld diameter and weld depth were measured thrice and average value was obtained for each set of experiments.

**Figure 8:**
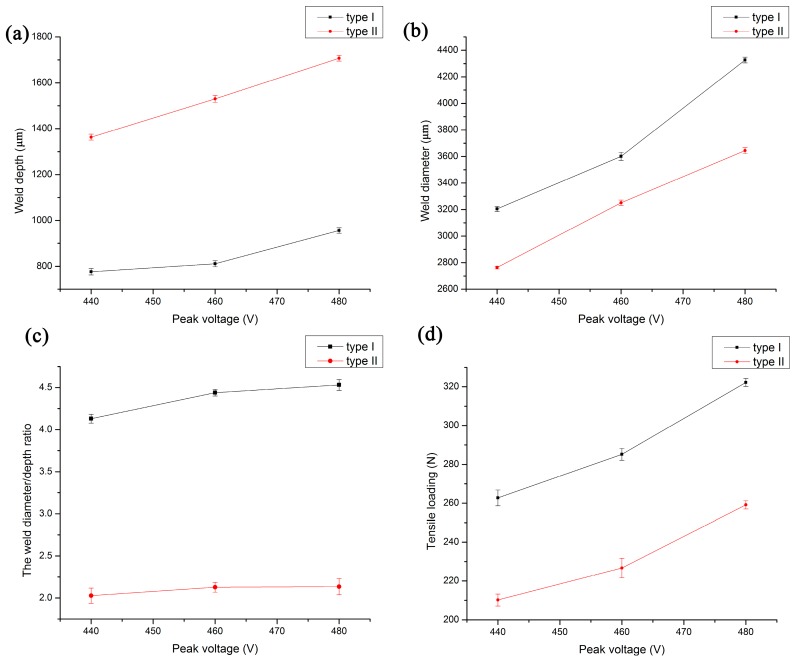
The effect of peak voltage on the welding conditions of type I and type II when defocusing distance is 8 mm. (**a**) Weld depth; (**b**) weld diameter; (**c**) the weld diameter:depth ratio; (**d**) tensile loading.

It is very important to improve the quality of the connection by studying the interactive effects of process parameters on the welding pool forming. Figure 8 shows the effect of peak voltage on the weld diameter, weld depth and the weld diameter:depth ratio of type I and type II, where the defocusing distance is 8 mm. As shown in Figure 8a,b, with the increase of the peak voltage, the weld diameter and weld depth gradually increase. However, under the same peak voltage, the weld depth of type I is significantly smaller than that of type II, while the weld diameter of type I is always greater than that of type II. This is because the action time of these two types is different, even though the total output energy of type I and type II is largely the same. The action time of type II is five times as much as that of type I. In addition, the upper and lower materials are the transparent PMMA. The absorption of laser energy mainly depends on the absorbent called Clearweld. Therefore, the melting part of type I mainly concentrates in the vicinity of the absorbent, which makes type I have a larger weld diameter. On the other hand, the materials in type II have enough time to melt through the heat conduction, which leads to a larger weld depth. It is found that the quality of connection is greatly affected by the weld diameter:depth ratio, which also has a certain relationship with the tensile load of the connecting piece. Figure 8c shows the relationship between the peak voltage and the weld diameter:depth ratio, where the weld diameter:depth ratio of type I is significantly larger than that of type II. However, the effect of peak voltage on the weld diameter:depth ratio is very small. As shown in Figure 8d, with the increase of the peak voltage, the tensile loads of type I and type II increase. Moreover, the tensile load of type I was greater than that of type II.

**Figure 9:**
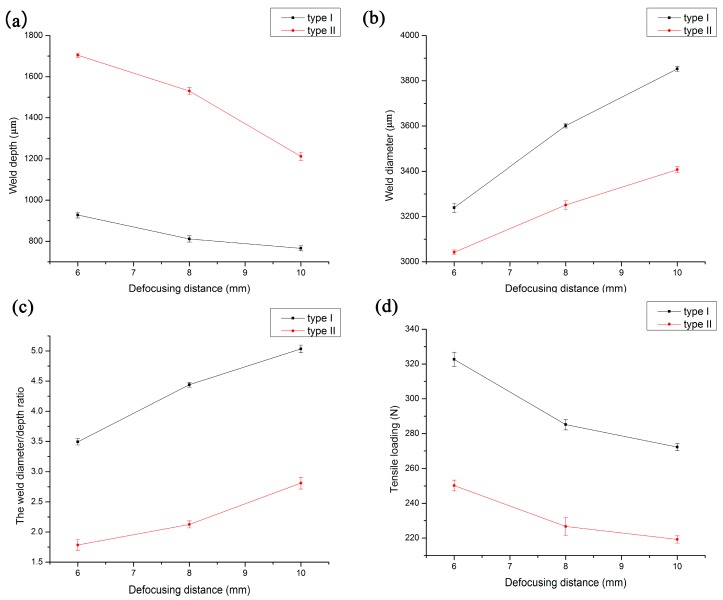
The effect of defocusing distance on the welding condition of type I and type II when peak voltage is 460 V. (**a**) Weld depth; (**b**) weld diameter; (**c**) the weld diameter/depth ratio; (**d**) tensile loading.

Figure 9 shows the effect of defocusing distance on the weld diameter, weld depth and the weld diameter:depth ratio of type I and type II, where the peak voltage is 460 V. As can be seen from Figure 9a,b, with the increase of defocusing distance, the weld depth decreases, while the weld diameter increases. Under the same defocusing distance, the weld depth of type I is less than that of type II, while the weld diameter of type I is always greater than that of type II. From Figure 9c,d, it can be seen that the tensile loads and the weld diameter:depth ratio of type I are greater than that of type II when the defocusing distance is the same. Nevertheless, with the increase of the defocusing distance, the tensile load decreases, while the weld diameter:depth ratio increases. In a certain range, the tensile loads decrease with the increase of defocusing distance, which has great influence on the morphology of weld pool and welding quality.

### 3.4. Temperature Distribution in Different Joining Types

Laser transmission welding is the process of rapid local heating and rapid cooling. Therefore, we can call laser transmission welding the nonlinear transient heat conduction process. Field function of the temperature field is a function of space and time domains. However, the space and time domains are not coupled. Thus, the finite element equation is established by the local discretization method.

The heat transfer mainly has three basic forms: heat conduction, heat convection and heat radiation. For the laser transmission welding of polymers, the main heat transfer form is heat conduction. Liao et al. [26] analyzed variations of shear strength that depend on the fiber laser process during micro-spot welding of AISI 304 stainless thin sheets. A preliminary study used ANSYS 12.0 results to obtain initial process conditions. The results show that the response surface methodology (RSM) and back propagation neural network/integrated simulated annealing algorithm (BPNN/SAA) methods are both effective tools for the optimization of micro-spot welding process parameters.

The thermal analysis model is established based on the volumetric heat source. According to heat transfer and energy conservation of Fourier’s law, the temperature field control equation of nonlinear transient heat conduction is given by [27]:

(2) $$Q\;=\rho (T)c(T)\left(\frac{\partial T}{\partial t}\right)-\lambda (T)\left(\frac{\partial^{2}T}{\partial x^{2}}+\frac{\partial^{2}T}{\partial y^{2}}+\frac{\partial^{2}T}{\partial z^{2}}\right)$$

where *Q* refers to the strength of the internal heat source; λ, ρ, and *c* refer to heat conduction coefficient, density, and specific heat of a material, respectively; and *T* and *t* refer to temperature and time variable, respectively.

As the material properties and the geometric condition of the object are known, the initial condition and the boundary condition are needed to obtain the special solution. The initial condition is

(3) $$T(x,y,z,0)=T_{0}$$

Among, (x,y,z)∈D, the boundary condition is

(4) $$k_{n}\frac{\partial T}{\partial n}-q+h(T-T_{0})+\sigma \varepsilon (T^{4}-T_{0}^{4})=0$$

where *D* refers to the model range; σ, *h* and ε refer to the coefficient of thermal radiation (5.67 × 10^−8^ W/m^2^·K), the coefficient of heat convection and the coefficient of thermal radiation, respectively; *T* and *q* refer to temperature and surface heat flux density, respectively. Due to the heat radiation condition, the temperature field distribution becomes a typical nonlinear problem.

**Figure 10:**
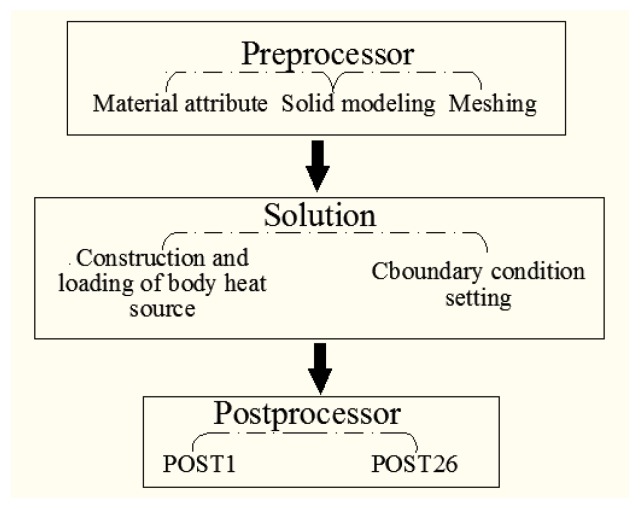
Simulation analysis process.

The change of temperature field and temperature time relationship of the highest point of PMMA is characterized by the thermal analysis model. The process can roughly be divided into: preprocess, solution and postprocessor, as shown in Figure 10. The physical performance parameters needed in the temperature field simulation are shown in Table 2.

**Figure 11:**
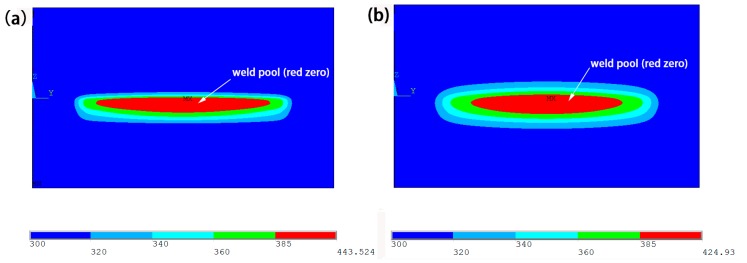
Comparison of weld pool when the peak voltage is 460 V and the defocusing distance is 8 mm. (**a**) type I; (**b**) type II.

Figure 11 shows the weld pool of *Y*–*Z* cross section of the upper and lower layers of the PMMA in type I and type II under the condition that the peak voltage is 460 V and the defocusing distance is 8 mm. The melting point of PMMA material is 385 K. When the temperature is higher than 385 K, the upper and lower layers of materials can form a weld. At the same time, the weld pool is formed inside the material. As can be seen from the diagram, the red area is the morphology of the weld pool, and the weld pool of type I is long and thin, but the weld depth of type II is larger than that of type I. The maximum temperature is different in the connection area for type I and type II. This is because the energy density is different for these two types. Since the peak voltage and the defocusing distance have been determined, the difference of pulse frequency and duration leads to different energies in the connection area. Therefore, the energy density of type I was much greater than type II. In addition, the temperature of type I was higher than type II.

## 4. Conclusions

This study assessed the efficacy of the welding types of PMMA using Clearweld as the absorbing medium. Three variables were investigated: peak voltages (440 V, 460 V, 480 V), defocusing distances (6 mm, 8 mm, 10 mm) and the welding type: type I (pulse frequency and the duration for 25 Hz, 0.6 s) or type II (pulse frequency and the duration for 5 Hz, 3 s). These two types all had good performance in welding, and the welding strength of type I was stronger than type II for PMMA.

The following conclusions can be made from the results:

(1) Laser transmission spot welding of type I produced a lot of small bubbles and some big bubbles. These bubbles made the upper and lower layers produce a micro anchor, improving the welding strength. However, type II produced few bubbles and the bubbles were very small.
(2) The welding strength of type I was higher than type II. In addition, the tensile failure mode of type I was the base sheet fracture along the solder joint. This indicated that the welding strength was higher than the tensile strength of the substrate. Furthermore, the tensile failure mode of type II is the interface failure.
(3) The weld pool diameter:depth ratio of type I was obviously larger than type II. The welding performance was better. It indicated that the weld pool diameter:depth ratio greatly affected the quality of the joint.
(4) From the result of thermal conductive analysis, heat was rapidly distributed throughout the same material of PMMA for the case of joints. Because the laser energy density of type I was higher than type II, the weld pool of type I is long and thin, and the temperature of type I was higher than type II.

## Figures and Tables

**Table 1:** Experimental parameters of spot welding.

Group	Sample Size (n)	Weld Type	Peak Voltage (V)	Defocusing Distance (mm)
1	3	type I	440	6
2	3	type I	440	8
3	3	type I	440	10
4	3	type I	460	6
5	3	type I	460	8
6	3	type I	460	10
7	3	type I	480	6
8	3	type I	480	8
9	3	type I	480	10
10	3	type II	440	6
11	3	type II	440	8
12	3	type II	440	10
13	3	type II	460	6
14	3	type II	460	8
15	3	type II	460	10
16	3	type II	480	6
17	3	type II	480	8
18	3	type II	480	10

**Table 2:** Physical performance parameters of polymethyl methacrylate (PMMA).

Material	Density [28] (kg/m^3^)	Specific Heat Capacity [28] (J/(kg·K))	Coefficient of Thermal Conductivity [28] (W/(m·K))	Viscous Flow Temperature (°C)
PMMA	1180	1470	0.21	220
